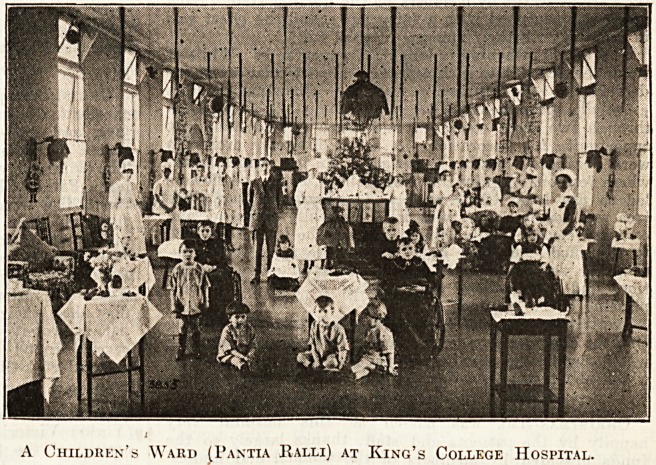# The Hospitals' Christmas

**Published:** 1921-01-08

**Authors:** 


### THE HOSPITALS' CHRISTMAS.

### THE LONDON HOSPITAL.

EVERY old Londoner who revisited his hospital this Christmas must have felt in his heart that the pre-war spirit of this season had truly returned. The opening function, the dress rehearsal, took place in the college library on Christmas Eve before an audience of sisters, students and their friends. Here all the *contretemps* that dog the footsteps of the ambitious were gone through, and seasoned warriors prophesied that the morrow's show would yet be a good one. Starting at 3.30 and lasting for two hours, a long procession of nurses and violinists, singing the sweetest of carols, wended its way very slowly and singing from ward to ward. The morning passed quickly, Christmas stockings were opened, letters from home were read and discussed, and Father Christmas, attended by a small host of courtiers and pages and fairies, visited every patient, his attendants leaving to each a present and to the nurses violets fresh from the fields.

Girl Students' Troupe at the London.

In Esther Ward at Guy's.

Although the House Committee had regretfully decided that funds would not run beyond beef, yet somehow Matron saw that every ward had its turkey, and these were dissected by the housemen, dressed as chefs, in their own wards. This year the decorations of the wards showed an element of novelty and ingenuity that was refreshing. One ward showed countless apes clustering among the traditional festoons of greenery; others were decorated with coloured balloons. One children's ward illustrated almost all the nursery rhymes in a most ingenious fashion. Great amusement was afforded by the residents' band, whose rehearsal is here illustrated, in pirate-like costume, playing on toy symphony instruments, and giving a mock recital of hospital noises and a typical parody of doctors' and nurses' woes. The college students and the lady students each ran a troupe, and among all, happy and spreading happiness, moved the House Governor and Mr. Paulin, the treasurer, trusting to God for next Christmas and thanking everyone, from the staff to the porters, for this day's happiness. In the receiving-room we all sang "Auld Lang Syne," and then moved to the out-patients' department for the maids' annual Christmas dance.

### THE ROYAL FREE HOSPITAL.

IT is an unerring fact that only the truly great can descend to the level of childhood, and to watch well-known men disporting themselves as "Santa Claus," giving up precious time to the call of the children, is to feel a pleasure that is akin to pain. Christmas was wonderful, and New Year's Day a great day, at the Royal Free Hospital. About three hundred invitations had been issued to the children who had attended as casualty and out-patients during the year that has just gone, and judging by the number that put in an appearance, most of the invitations had been accepted. At 3.30 the little people began to arrive, and soon—very soon—they were squeezing up tight against one another, waiting for the moment when the signal would be given for them to begin. At last Matron (Miss Cox-Davies) gave out the "grace" to sing, and little voices joined timidly in the song. And then tea began. Such an onslaught on the cakes, such a noise of bursting crackers, such a dressing-up in paper hats, and such a blowing of wooden whistles—surely a noise for all childhood to revel in! For an hour the eating and drinking went on. Prince Charming wandered up and down looking for his lost Princess, and Pierrot and Columbine waxed merry with the children. When at last no one could eat any more, the tables were cleared, the forms all turned one way to face the stage, and the entertainment began. Hearty congratulations to the students, who presented an entirely new and impromptu version of "The Sleeping Beauty," and special mention

Rehearsing at the London.

**The Hospitals' Christmas**—*(continued)*.

to the little "Jester" of the play. And then came a Pierrot troupe, composed of students again. With dance, recitation, and song they swung into popularity, and it was with little alacrity the children would let them go when they sang their final song, "Good-night."

But that was not the end. The lights were lowered and the children bidden by Miss Cox-Davies to keep quite still and listen for the knock of strangers who had come from far away. "Bang, bang, bang," at the door, and the roof nearly fell in with the shout of "Come in." And in came Santa Claus, or two "Santa Clauses," as one little boy called out, Dr. Phear and Mr. Joll, honorary physician and surgeon to the hospital. Such a wild, wonderful clamour, such happiness and sheer joy! Little ears could scarcely listen to Father Christmas speaking, and little feet scarcely could be persuaded to wait their turn. But each little person stole round at last, and was made happy by a gift from the tree.

And when at last, tired, excited, exhausted, and happy, the children took their leave, one gazed across the fast-emptying hall and felt glad of the privilege of being a looker-on.

### THE LONDON HOMŒOPATHIC HOSPITAL.

The wards were this year made even more attractive than usual, for loving hands had woven tasteful decorations all through the building, while scores of fairy lamps illuminated the wards. A huge Christmas-tree was placed in the children's ward, and the annual distribution of gifts to the patients and nurses was an occasion of many of the hospital's supporters to pay a visit of inspection. There was a great gathering in the wards, and one of the doctors, in traditional guise, acted as Father Christmas. For the elder patients there were different articles of wearing apparel, and for the children many kinds of toys, etc., all given to the hospital. Nor were the nurses forgotten, for they participated in the "fruit" of the magic tree. So far as staff and visitors were concerned, the greatest fun began with the distribution of the annual good-humoured "jokes," which the members play on each other by way of limericks, couplets, and verses, and other means, in which the eccentricities and foibles of the recipients are jokingly dealt with. The reading of these "gifts" was accompanied by great merriment.

### ADDENBROOKE'S HOSPITAL, CAMBRIDGE.

At no hospital in the land is the spirit of Yuletide more manifest than at Addenbrooke's, but Christmas 1920 has truly transcended all others. Happiness and mirth seemed to abound everywhere. The good time secured was due in no small measure to a jumble sale, organised early in November by the Matron, Miss Crookenden, and a band of willing helpers, realising the splendid sum of £67 for Christmas funds.

On Christmas Eve the old-time custom was carried out. The Matron and the nursing staff and the choir of Great St. Mary's Church went round the wards singing Christmas carols. At 6 o'clock on Christmas morning the majority of the nursing staff attended service at Little St. Mary's Church. Preparations for Christmas dinners occupied most of the morning, and at noon in were borne the steaming turkeys, vegetables, and plum-pudding, followed by dessert and an abundance of crackers. During the afternoon each ward was visited by the Mayor, and excellent music was provided by an orchestral band.

Sumptuous teas were laid throughout the Hospital, each patient being allowed to invite friends, and the domestic staff, a very important wheel in the machinery of the Institution, were by no means forgotten, and they had their Christmas dinner at 1.30 on Saturday, with the usual good things.

At 5.30 all who could walk or be wheeled made their way to the large out-patient hall to enjoy the splendid concert which had been arranged, and a most delightful evening was spent. The decorations, always a strong feature, were especially good this year.

Sunday was spent quietly. On Monday (Boxing Day) the nursing staff had their Christmas dinner in the out-patient hall, which was beautifully decorated with Chinese lanterns and fairy lights, and seemed a veritable fairyland, the table being laden as usual with the handsome silver, which is always sent by Trinity College; and then a surprise for the nurses! They saw a Christmas-tree brilliantly illuminated, and discovered to their joy that the Matron was distributing the many coloured parcels to her nursing staff, who were much pleased with their useful gifts. Afterwards there were games, and a few dances until 10 P.M.

Tuesday, the 28th, was the Children's Day, when 120 out-patient children assembled for tea and the Christmas-tree. On Wednesday, the 29th, the Matron was at home to all the resident nursing and domestic staff.

### THE QUEEN'S HOSPITAL, BIRMINGHAM.

A very happy round of Christmastide events was most appropriately commenced on Christmas Day by a tour of the wards by the Lord Mayor (Alderman W. A. Cadbury) of Birmingham and the Lady Mayoress, accompanied by the Chairman (Brigadier-General Sir John Barnsley), several other members of the Committee, Drs. Purslow and Thomas, the Chaplain (the Rev. G. H. Moore), the matron (Miss E. Bullivant), and the General Superintendent (Mr. Hedley Lucas).

The wards had been most tastefully decorated by the sisters, nurses, and kind helpers, and presented an excellent appearance. The resident medical staff (including three ladies), cleverly garbed as a policeman, Quakeress, sailor, sailor's daughter, monkey, costers, and "bookie" respectively, created considerable amusement by a fancy-dress parade.

In the out-patient department an animated and interesting scene was witnessed as Father Christmas, in the person of the casualty house surgeon, assisted by the sisters and nurses, handed toys, etc., from the fully-laden Christmas-tree to the all-expectant children. Later Father Christmas, together with the matron and the general superintendent, visited the wards, and each patient was given useful presents. Christmas fare was partaken of, and happiness was supreme.

In the evening a most enjoyable entertainment was given by the sisters and nurses; and on Boxing night the resident medical officers delighted their many hearers with a concert comprising vocal and instrumental music, a duologue, and a recitation, all excellently given.

### THE BROMPTON HOSPITAL FOR CONSUMPTION.

Christmastide and New Year's Eve festivities ended at this well-known charity with a substantial tea to some hundreds of children from the out-patients' department given in the spacious entertainment hall of the hospital, beautifully decorated with evergreens, coloured lights, etc., followed by a Punch and Judy show and a huge Christmas-tree with a present for each little visitor. Father Christmas, in the person of Dr. L. S. T. Burrell, one of the physicians to the hospital, appeared on the scene, his carriage being drawn by a most life-like stage donkey, impersonated by two of the house doctors.

**The Hospitals' Christmas**—(*continued*).

Through the kindness of members of the Committee and many friends, a most generous fare was provided for the patients on Christmas Day. The Chairman (Lord Cheylesmore), Mr. G. A. MacMillan, Mr. Cohen, the Chaplain (the Rev. Harvey Grindon), members of the visiting medical and surgical staff, and many others assisted to help make glad the hearts of our suffering brothers and sisters in the name of the Great Physician.

Father Christmas was very busy quite early and, laden with gifts, paid a visit to each and every ward, where by this hour there was much mirth and merriment. Whilst all this was going on the kitchen staff were kept very busy indeed in preparing for the dinner, which brought all manner of good things for those unable to partake of the richer part of the day's dietary.

TODD MATERNITY WARD (KING'S COLLEGE HOSPITAL).

Nor must the indefatigable efforts of the matron and the nursing staff be forgotten, and by whom carols were given. In the afternoon six concert parties toured the wards, and it was universally agreed that no more successful and happy Christmas had ever been spent at this hospital.

### KING'S COLLEGE HOSPITAL.

FOR several months prior to the advent of this great festival the one thought which dominated the minds of the whole staff at King's College Hospital was how best to make the patients as happy as possible, and, as will be seen from the following account, no stone was left unturned towards attaining this end.

The hospital choir struck the first note of Christmas-tide carrying the message of the birth of the Saviour of mankind to each of the fifteen wards in this wonderful house of healing. A special visit was paid to the children's ward, where was held a Christmas service, and to hear those little ones as they lay in their cots lisping "Once in Royal David's City" truly touched the heart.

Services were held at 6 A.M., 8 A.M., 10 A.M., and 5.30 A.M.; 180 people, consisting of members of the staff, nurses, doctors, and patients, made their early morning communions, those who were unable to be present in the chapel being attended to at the bedside between the hours of 7 and 8 A.M.

At the evening service special carols were sung by soloists and choir, after which, with His Benediction ringing in our ears, all went forth inspired and encouraged

The description of a few of the ward decorations which follows will give some idea of the vast amount of time and labour which was devoted by the ward sisters and nurses, and one does not hesitate to say a labour of love for the patients.

PANTIA RALLI (CHILDREN'S WARD).

The ward was gay with coloured shades, balloons and flying birds were hung from the ceiling, but the chief attraction was the splendid Christmas-tree, laden with toys of all sorts, for the children of the ward and for small patients invited from the other wards of the hospital.

LISTER (WOMEN'S SURGICAL).

Water-lilies as lamp shades—pale colours—with butterflies alighting on plants, flowers, and foliage. A Christmas-tree, with a realistic snow scene from one of the Norwegian (?) Christmas tales, decorated the fire-place at the children's end of the ward.

LONSDALE (MEN'S SURGICAL).

The ward was decorated entirely with red and with evergreens, lamp-shades in red, and the three red balloons hanging from the centre lights caused great amusement among the patients, who thought they were meant to represent the "three balls." There was great excitement when the nigger troop with the jazz band visited the ward on the day of the concert.

A CHILDREN'S WARD (PANTIA RALLI) AT KING'S COLLEGE HOSPITAL.

TODD (MATERNITY WARD).

The ward was gaily decorated with oranges and lemons festooned from side to side. On one of the fireplaces was

**The Hospitals' Christmas—(*continued*).**

a seashore scene—babies floating to land on waterlilies, welcomed by a sailor from H.M.S. *Iron Duke*. From beneath each lampshade there peeped a baby's face. To this ward were welcomed former "Todd babies," who thoroughly enjoyed a good tea and the gifts from the splendid Christmas-tree in the middle of the ward. Great was the fuss made of these small visitors by the pupil midwives.

The Sister Matron, Assistant Matron, Sister Housekeeper, all the various sisters, nurses, resident medical officers, medical students—in fact, the whole staff—laboured unceasingly and ungrudgingly that the season of Christmas might be made one of great joy and happiness.

Great appreciation has been expressed on every hand of the great and unselfish devotion exhibited by every member of the staff in the best interests of the patients and for their happiness.

The cost of the season's festivities has been defrayed from funds specially raised for the purpose, so that there has been no charge on the general funds in any way.

### THE GREAT NORTHERN HOSPITAL.

Lord Islington and Mrs. Alfred Barratt presented Christmas-trees to the Children's Wards, and "Santa Claus" gave every tiny patient a gift on Boxing Day. On Christmas Day a special dinner was provided, and every adult patient allowed to invite a friend to afternoon tea. Carol-singing by the choirs of the Presbyterian Church, Crouch Hill (conductor, Mr. Harold Bristol), and St. Michael's, Highgate (conductor, Mr. Pinches), took place in the wards on Christmas Day. Excellent arrangements to celebrate Christmas were made by all sections of the hospital.

The expenses were defrayed by contributions received for the purpose, and Christmas festivities at the Reckitt Convalescent Home, Clacton-on-Sea (a branch of the Great Northern Hospital), included a Christmas-tree for children, a whist-drive for adults, and a special dinner for all patients, the expenses here being met by local residents.

### THE GENERAL HOSPITAL, WOLVERHAMPTON.

The Christmas celebrations at the hospital commenced at 9 p.m. on Christmas Eve with the singing of carols in the various wards by members of the nursing staff. Each nurse was equipped with a fairy lamp, and the effect was in keeping with their message of good will.

The wards presented a delightful appearance. While it would be difficult to pick out any one particular ward for description, the children's ward at Christmas-time is especially prominent. The approach to this ward is through two short corridors, and these offered abundant opportunity for decoration. A series of pretty archways were arranged, leading eventually to the large ward, where festoons of gaily-coloured ribbons, flags, and flowers led to the huge Christmas-tree in the far corner. The tree was heavily laden with toys, tastefully arranged, and coloured electric lights shining through the branches added to the effect. Another ward which calls for special mention was decorated by Chinese lanterns of every description, and when these were lighted at eventide a very pleasing effect resulted.

Festivities were continued during the week. On Boxing Day the chief event was the nurses' fancy-dress dinner, in which about sixty members of the staff took part. It was which about sixty members of the staff took part.

### CHELSEA HOSPITAL FOR WOMEN.

Christmas-time was passed at this hospital very happily by the patients and staff, thanks largely to the funds provided by members of the Council and Ladies' Committee. The celebrations included carol-singing by members of the staff and by the choir of St. Peter's, Cranley Gardens; Christmas dinners for patients and nurses, the latter in fancy dress; special teas for patients and their friends; an entertainment by the staff on Boxing Day, and another, partly theatrical, by the members of the Chelsea Nurses' Club.

### LONDON TEMPERANCE HOSPITAL.

Christmas Day was observed at the London Temperance Hospital in a manner which will not soon fade from the memory of the patients who spent it there. The wards were beautifully decorated with original designs, which transformed them into bowers of flowers, with delicate green festoons and shaded electric globes of every hue. At 8 a.m. the matron, sisters, and nurses visited each ward and sang some of the exquisite old Christmas carols. At 12 noon a dinner of turkey, plum pudding, mincepies, fruit, and dessert was served, with special delicacies for those who were too ill to enjoy such a repast. At 3 o'clock visitors were admitted, and teas were served all the afternoon in the sisters' sitting-room and in the wards. During the afternoon the senior house surgeon (Mr. Shirley Fawsett), as "Father Christmas," visited the patients and distributed presents; so splendid was his impersonation that no one recognised him, and the shouts of glee with which he was greeted in the children's ward was a most refreshing change in these days of pessimism and materialism.

A huge fir-tree, perfect in its symmetry, having been sent all the way from the North of Scotland, was erected in the children's ward, and literally groaned under its burden of toys and garments. The children treated in this hospital are drawn from a very poor neighbourhood, and their delight was simply overwhelming.

### LORD MAYOR TRELOAR CRIPPLED CHILDREN'S HOSPITAL.

The Christmas festivities opened at the Lord Mayor Treloar Crippled Children's Hospital by the carol-singers (members of the nursing staff) visiting all the wards on Christmas Eve, wearing their quaintly-hooded uniform cloaks and carrying multi-coloured lanterns swinging from the end of a long pole.

Christmas Day opened with a celebration of Holy Communion in the chapel at 6.30 for the staff, and the business of the day began by the distribution of stockings—some of which were bed-socks and held a lot. The wards vied with one another in schemes of decoration. There were Christmas-trees laden with toys for all, ready for the distribution, after dinner, when Father Christmas (Sir Henry Gauvain) and his fairy (Dr. C. E. M. Jones) came round the wards, both bearing large boxes of chocolates, the gift of Queen Alexandra, the distribution of which took precedence to the distribution of the toys from the trees. Next came troops consisting of members of the nursing staff, made up as golliwogs and rag dolls, who gave charming little entertainments of song and dance, to the immense delight of the children.

### ROYAL VICTORIA HOSPITAL, FOLKESTONE.

Christmas spent in a real old-fashioned way. Most excellent dinner of turkey, Christmas pudding, mince pies, and fruit; concert and games after. Each patient had a parcel of useful gifts sent by the Kent V.A.D. The children's ward was most beautifully decorated with a large Father Christmas in place of the usual Christmas-tree. The wards were prettier than ever, the sister on the male floor chose crimson and the female floor had pink with a border of black. On Wednesday the male floor concert was given, the "Rhapsodies" from the Pavilion kindly giving a most excellent entertainment. The final festivity was on Thursday, when the female floor had their concert and Christmas-tree, when each patient received a gift and a dip from a lucky bag carried round by Father Christmas. It was altogether a wonderful Christmas here.

### THE KEIGHLEY VICTORIA HOSPITAL.

Christmas was celebrated most heartily at the Keighley (Yorks) Victoria Hospital, and for some time before the arrival of the festive season Miss Garner (the Matron), who had the keen co-operation of the staff, had been considering the most suitable method of celebrating the occasion, and the result of her efforts was highly gratifying in every way. The various wards had been tastefully decorated, evergreens and freshly cut flowers being very prominent, while there were exquisite paper decorations—chains, flowers, and festoons—the staff of each ward vying with each other in eclipsing the beauty and charm of the respective wards. Perhaps the most beautiful spot was the nursery, the room having been transformed into a veritable Fairyland.

## Figures and Tables

**Figure f1:**
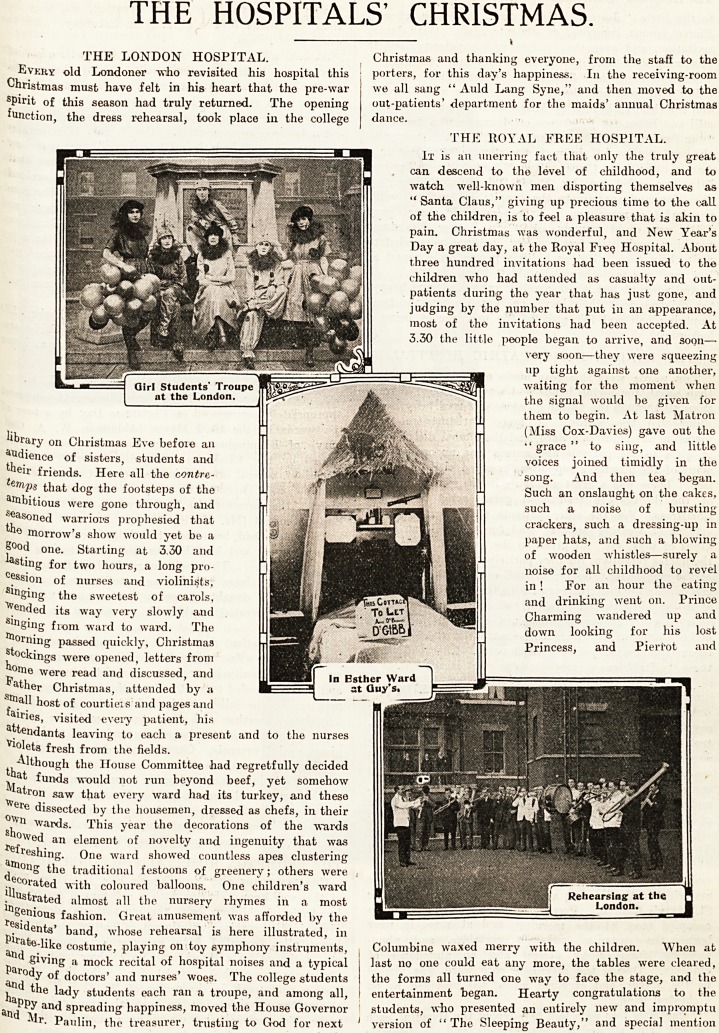


**Figure f2:**
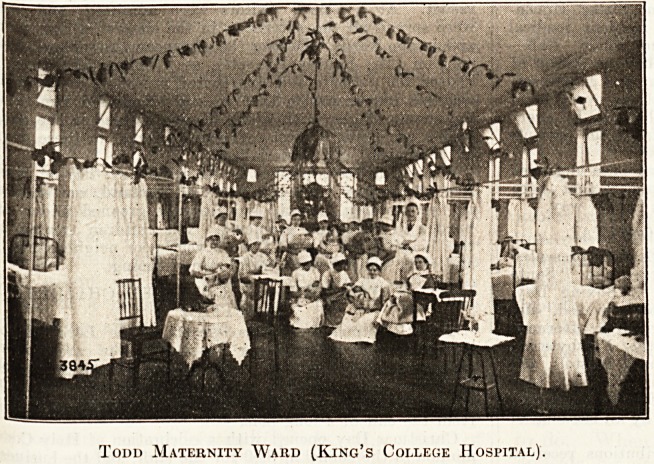


**Figure f3:**